# Reciprocal regulation of cellulose and lignin biosynthesis by the transcription factor OsTCP19

**DOI:** 10.1093/jxb/erad408

**Published:** 2023-12-20

**Authors:** Konan Ishida

**Affiliations:** Department of Biochemistry, University of Cambridge, Hopkins Building, The Downing Site, Tennis Court Road, Cambridge CB2 1QW, UK

**Keywords:** Biomass, grain yield, growth penalty, lodging resistance, plant cell wall, transcription factor

## Abstract

This article comments on:

Lv S, Lin Z, Shen J, Luo L, Xu Q, Li L, Gui J. 2024. *OsTCP19* coordinates inhibition of lignin biosynthesis and promotion of cellulose biosynthesis to modify lodging resistance in rice. Journal of Experimental Botany 75, 123–136.

**Cultivation of semi-dwarf varieties of cereal plants brought about a dramatic improvement in grain yields due to reduced lodging risk under high fertilization conditions. Lodging resistance is also a useful trait to select promising cultivars. However, semi-dwarf varieties tend to have negative effects on reproductive organ size and limit plant biomass. Hence, new approaches are required to fine-tune the mechanical properties of cereal plants to overcome this trade-off. Lv *et al*. (2024) revealed that the transcription factor OsTCP19 promotes cellulose biosynthesis and inhibits lignin biosynthesis simultaneously, which contributes to mechanical strength. The fibre cell-specific overexpression of *OsTCP19* achieved higher lodging resistance without compromising grain yield per plant, thus overcoming the negative trade-off observed in plants with constitutive *OsTCP19* overexpression. These results provide a novel genetic engineering approach towards sustainable food production.**

The rise of civilizations was accompanied by the domestication of plants with useful traits for human use. In typhoon-prone East Asia, there has been a need for rice plants that are resistant to lodging, which is defined as the permanent displacement of above-ground parts from their upright position as a result of stem buckling and/or root displacement. The ability of plants to withstand lodging is thus governed by the physical strength of their stems, or culms in the case of cereal, which is tightly linked to the cell wall composition. Indeed, many of the genes responsible for ‘brittle rice mutants’ are cell wall related (Table 1; Fig. 1). Given the negative relationship often observed between lodging resistance and grain yield, there is a need to understand the molecular mechanisms of cell wall biosynthesis genes in order to engineer cell walls with higher flexibility.

**Fig 1.**
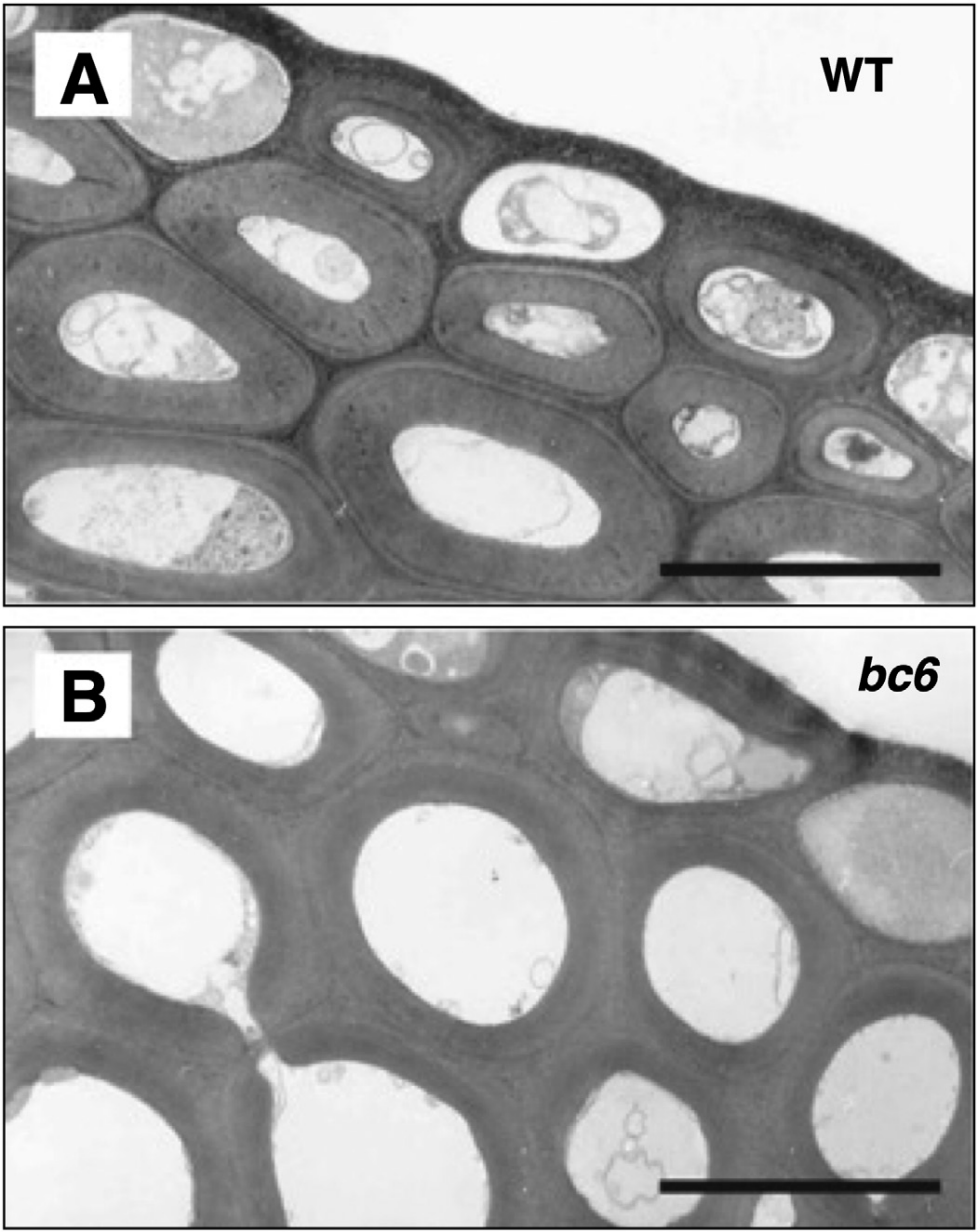
Electron microscope image of *brittle culm6*. Most of the *brittle culm (bc)* mutants listed in Table 1 showed different cell architecture. For instance, the *bc6* mutant had round cells that lacked cell corners. WT: Taichung 65 cultivar. Scale bar=5 μm. Image courtesy of Dr Toshihisa Kotake.

**Table 1.** Mechanical traits are often linked to plant cell wall properties

Mutant name	Responsible gene	Reference
*brittle culm 1*	GPI-anchored protein	Sato *et al*. (2010); Liu *et al*. (2013)
*brittle culm 3*	Dynamin	Hirano *et al*. (2010)
*brittle culm 6*	CESA9	Kotake *et al*. (2011)
*brittle culm 7*	CESA4	Yan *et al*. (2007)
*brittle culm 10*	Unclassified glycosyltransferase-related to AGP (DUF266)	Zhou *et al*. (2009)
*brittle culm 11*	CESA4	Zhang *et al*. (2009)
*brittle culm 12*	Kinesin	Zhang *et al*. (2010)
*brittle culm 13*	CESA9	Song *et al*. (2013)
*brittle culm 14*	Nucleotide sugar transporter	Song *et al*. (2011)
*brittle culm 15*	Chitinase	Yi *et al*. (2022)
*brittle culm 16*	GPI-lipid *O*-acetyltransferase	Xu *et al*. (2022)
*brittle culm 18*	Xylan synthase (IRX10)	Wang *et al*. (2022)
*brittle culm 19*	CESA4	Ma *et al*. (2021)
*brittle culm 24*	UDP-glucose epimerase	Zhang *et al*. (2020)
*brittle culm 25*	UDP-xylose synthase	Xu *et al*. (2023)

Mechanically weak rice plants have been isolated by forward genetic screening. Many of the causal genes are related to cell wall biosynthesis. In particular, mutations in *CELLULOSE SYNTHASE* (*CESA*) are influential. Others include the cytoskeleton and COBRA-like GPI-anchored proteins known to affect cell morphology in Arabidopsis. AGP, arabinogalactan protein; DUF, domain of unknown function; IRX, irregular xylem.

Plant mechanical strength comes from the rigidity of the secondary cell wall, composed primarily of cellulose and lignin. Secondary cell wall synthesis is induced in a hierarchical manner: the VASCULAR-RELATED NAC-DOMAIN transcription factor family is the master regulator, with Tier 2 transcription factors (e.g. MYB46 and MYB83) at an intermediate level. Downstream Tier 3 transcription factors (e.g. MYB4 and MYB58) regulate the expression of multiple sets of cell wall-related genes. Divergent regulatory pathways are thought to enable tissue- and condition-specific cell wall control (Rao and Dixon, 2018). Cellulose and lignin are both essential elements of the cell wall, but there are several known examples of reciprocal synthesis (e.g. simultaneous suppression of 4-coumarate-CoA ligase and coniferaldehyde 5-hydroxylase decreases lignin content and increases cellulose content) (Li *et al.*, 2003). However, why and how the opposite regulation is caused is still waiting to be elucidated.

Lv *et al*. (2024) found that the transcription factor TEOSINTE BRANCHED1-CYCLOIDEA-PCF 19 in rice (OsTCP19) modulates cellulose and lignin in an opposite manner via two underpinning transcription factors (Fig. 2). First, the authors manipulated the OsTCP19 activity by fusing a repression or activation module on OsTCP19 in rice. While the activation line became physically stronger compared with the wild type, the suppression line became weak against physical force (Table 2). In the field, lodging occurrence decreased by ~35% in the activation line but increased by 40% in the suppression line. The authors then analysed the chemical composition of the transgenic plants. Interestingly, cellulose contents were increased in the activation line, but lignin was less accumulated compared with the wild type. Based on this observation, the authors hypothesized that OsTCP19 induces cellulose biosynthesis genes but suppresses lignin-related genes. To test this hypothesis, they conducted transcriptome analysis using both lines. More than 1500 genes were identified as differentially expressed genes (DEGs). The breakdown of DEGs is dominated by those related to phenylpropanoid synthesis, which overlaps with the lignin metabolism pathway. Furthermore, the OsTCP19 activation line showed higher expression of cellulose biosynthesis genes, and lignin-related gene sets were suppressed. These results indicate that the lodging resistance improvement requires a specific pattern of secondary cell wall synthesis, with an increase in cellulose and a decrease in lignin.

**Fig 2.**
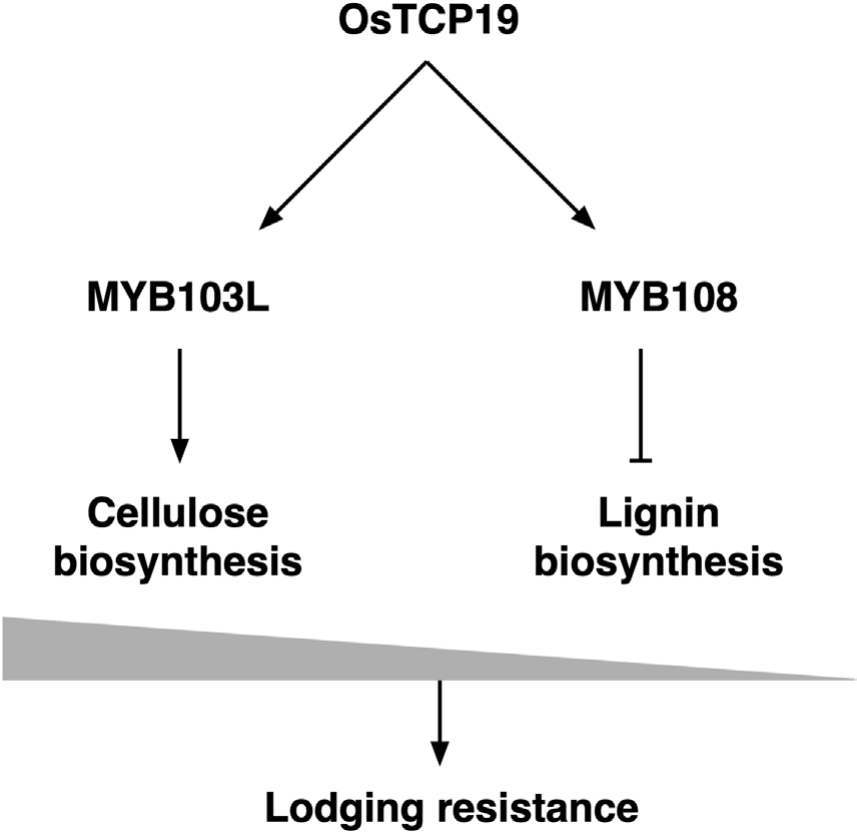
Proposed mechanism of *OsTCP19* function. OsTCP19 binds to the promoter regions of *MYB103L* and *MYB108*. *MYB103L* induces cellulose biosynthesis genes. *MYB108* suppresses lignin biosynthesis genes. This fine-tuned balance leads to higher lodging resistance (based on Lv *et al.*, 2024).

**Table 2.** Phenotypic comparison of *OsTCP19* transgenics

	Activation line	Suppression line	Fibre-specific activation line
Cellulose	12% increased	10% decreased	13% increased
Lignin	20% decreased	15% increased	20% decreased
Mechanical strength	Stronger	Weaker	Stronger
Single plant grain yield^*a*^	>30% decreased	Slightly increased	Unchanged

Systemic activation of *OsTCP19* fell into yield decrease due to the trade-off. However, driving by a fibre-specific promoter enables mechanical strength and normal grain yield.

^*a*^ Observed under greenhouse conditions.

To reveal how cellulose and lignin synthesis are regulated in a reciprocal manner, the authors looked for the highly affected Tier 3 transcription factors from RNA-seq data. Together with the literature, *MYB103L* was identified as the modulator of cellulose biosynthesis, and *MYB108* as the candidate for lignin modulation. To obtain direct evidence that OsTCP19 controls their expression, a gel shift mobility assay was performed using recombinant OsTCP19 and the promoter regions of *MYB103L* or *MYB108*. OsTCP19 could directly bind to both regions. Together with the results of the transcriptional analysis, the authors deduced that *OsTCP19* has dual activity as a positive regulator for cellulose biosynthesis and a negative regulator for lignin biosynthesis.

Distinctive sclerenchyma structures in lodging-resistant rice varieties have previously been identified (Zhang *et al.*, 2021). As mentioned, the constant overexpression line of OsTCP19 reduced lodging but also reduced yield. Therefore, Lv *et al*. (2024) tested tissue-specific *OsTCP19* expression using a sclerenchyma fibre cell-specific promoter. Tissue-specific expression lines showed no obvious change in appearance (plant height and tiller number) but increased mechanical resistance (Table 2). Chemical composition in mature stems supports that this promising phenotype came from the increase of cellulose and decrease of lignin the same as the *OsTCP19* activation line. Interestingly, the tissue-specific expression line did not show a decrease in individual plant grain yield, overcoming the trade-off between growth and physical strength. It will be important, however, that further outdoor trials are conducted to understand if this relationship remains in nitrogen-rich paddy fields.

In a wider context, this study highlights the importance of engineering only those parts of the trade-off that are linked to agriculturally useful traits. In this example, while fibre cells are the key for mechanical strength, they cause a reduction in grain volume in seeds, which could be solved by changing the expression profile. In other words, the key to selective breeding is to identify and eliminate the causes of the unfavourable phenotype.

An open question relevant to the current study is why an increase in cellulose and a decrease in lignin change cell wall properties. It is also unknown why fibre cells play a major structural role. Furthermore, the effects of other TCP family members on cell wall synthesis remain to be elucidated.
